# Intraosseous Inflammatory Myofibroblastic Tumor of the Posterior Mandible: A Rare Case with Immunohistochemical Interpretation 

**DOI:** 10.30476/dentjods.2024.103119.2422

**Published:** 2025-12-01

**Authors:** Vandana Pandey Tripathi, Shubhangi Mani, Amit Mani, Manas Bajpai

**Affiliations:** 1 Dept. of Pedodontics and Preventive Dentistry, Government Dental College, Mumbai, India.; 2 Dept. of Orthodontics and Dentofacial Orthopaedics, Rural Dental College, Loni (Maharashtra), India.; 3 Dept. of Periodontology, Rural Dental College, Loni (Maharashtra), India.; 4 Dept. of Oral Pathology and Microbiology, Rural Dental College, Loni (Maharashtra), India.

**Keywords:** Inflammatory pseudotumor, Mandible, Tumor, Jaw

## Abstract

Inflammatory myofibroblastic tumor (IMT), previously called an inflammatory pseudotumor, is an unusual, rare, benign, but aggressive tumor of soft tissue origin. It is histopathologically characterized by heterogeneous proliferation of myofibroblasts with admixture e of various inflammatory cells, including lymphocytes, plasma cells, histiocytes, and eosinophils. IMTs are rare in the head and neck region and are exceedingly rare in the jaws as central lesions; only nine cases have been reported in the literature. We report a case of intra-osseous IMT occurring in the posterior mandible of a 28-year-old lady with a detailed discussion of treatment, histopathological, and immunohistochemical features.

## Introduction

Inflammatory pseudotumor was the term given to various neoplastic and non-neoplastic entities with common histopathological aspects, including spindle cell proliferation and chronic inflammatory cell infiltration composed of lymphocytes, plasma cells, eosinophils, histiocytes, etc. [ 1 ]. The first case of inflammatory myofibroblastic tumor (IMT) was described by Brunn in 1939 [ 2 ]. Various terminologies have been used to designate this tumor, including inflammatory pseudotumor, plasma cell granuloma, fibrous xanthoma, and pseudosarcoma, until the WHO in 1994 designated it as IMT [ 3 ].

IMT is a rare tumor that mostly occurs in the lungs, liver, and orbit. In the head and neck region, reports of IMTs have been found in the parapharyngeal spaces, maxillary sinus, epiglottis, salivary glands, and oral cavity [ 4 ]. However, IMTs occurring in the jaw bone as an intraosseous lesion are an extremely rare phenomenon. IMTs tend to imitate malignant lesions on clinical and histopathological parameters [ 3 - 4 ]. An exhaustive literature review has revealed only 14 cases reported previously; hence, the present case is preferably the 15th presentation of intra-osseous IMT.

## Case Presentation

**Figure 1:**
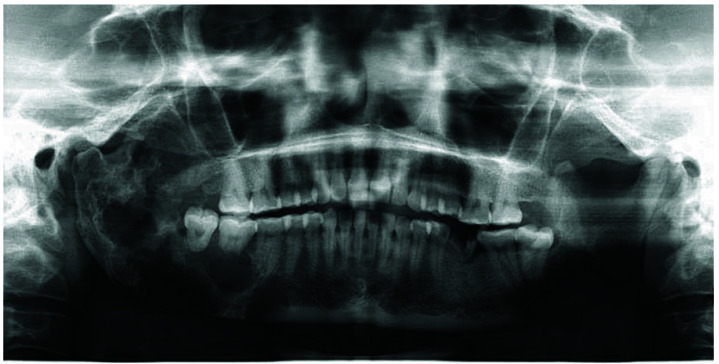
Panoramic radiograph of the patient

**Figure 2:**
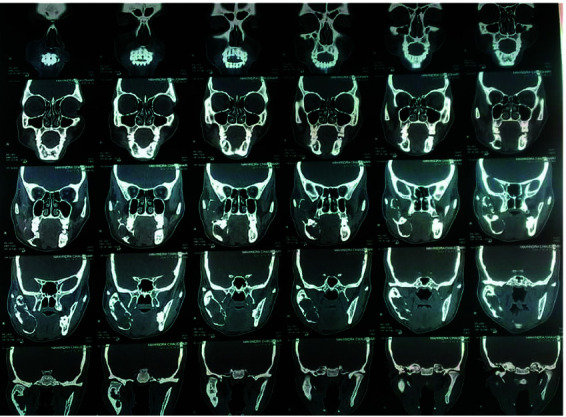
CT scan of the patient

An otherwise healthy 28-year-old lady presented to our teaching hospital for the evaluation of a localized, painless swelling on her right back region of the jaw from 5 months. The swelling was initially small and gradually reached its current size. The past medical history and family history were not contributing to the presenting symptoms. Past dental history revealed that the patient had undergone an extraction of tooth number 36 due to dental caries in her village. Intra-oral examination revealed a hard bony swelling on the right posterior mandibular region with relation to teeth numbers 45-48. The overlying mucosa was normal in color without showing any signs of inflammation or sinus formation. The cervical lymph nodes were not palpable. A panoramic radiograph showed a large, multilocular, osteodestructive lesion extending from the root of 46 towards the posterior mandible to the ramus area. Root resorption of 47 and 48 was noted (Figure 1). The computed tomographic scan revealed a large expansile lesion of the right posterior mandible (Figure 2). 

The lesion was provisionally diagnosed as ameloblastoma. An incisional biopsy was taken, and the tissue was sent to the Department of Oral Pathology for histopathological evaluation.

**Figure 3:**
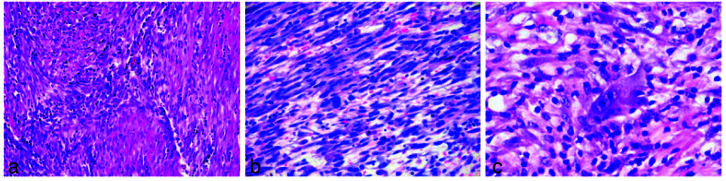
**a:** Fascicles of spindle-shaped cells with hyperchromatic nuclei in a hyalinized connective tissue stroma. (Hematoxylin and Eosin stain 20×), **b:** Plump and elongated spindle cells with hyperchromatic nucleus (Hematoxylin and Eosin 40 ×), **c:** Inflammatory cell infiltration composed of lymphocytes, plasma cells and eosinophils (Hematoxylin and Eosin stain 40×)

The microscopic examination of a hematoxylin and eosin-stained soft tissue section revealed fascicles of spindle-shaped cells with hyperchromatic nuclei in a hyalinized connective tissue stroma (Figure 3a). The spindle cells were plump and elongated, and the hyperchromatic nucleus showed minimal dysplasia (Figure 3b). The sheets of spindle cells were interspersed with an inflammatory cell infiltrate composed chiefly of lymphocytes and plasma cells, along with histiocytes and eosinophils (Figure 3c). On the basis of histopathological features, a diagnosis of an intermediate-grade spindle cell tumor with inflammation was made. Immunohistochemistry was performed using Vimentin, anaplastic lymphoma kinase (ALK), smooth muscle actin (SMA) S-100, Desmin. 

**Figure 4:**
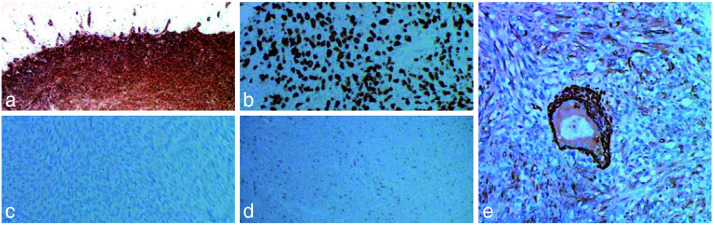
**a:** Diffuse positive expression of the tumor for Vimentin, **b:** Positive expression of the tumor cells for ALK, **c:** Negative expression of the tumor for S-100, **d:** Weak positive expression of the tumor cells for Desmin, **e:** Positive expression of the tumor cells for SMA (ALK: Anaplastic Lymphoma Kinase, SMA: Smooth Muscle Actin)

The spindle-shaped cells showed positive expression for Vimentin, SMA, and ALK, negative expression for S-100, and weakly positive expression for Demin (Figures 4a-e). 

**Figure 5:**
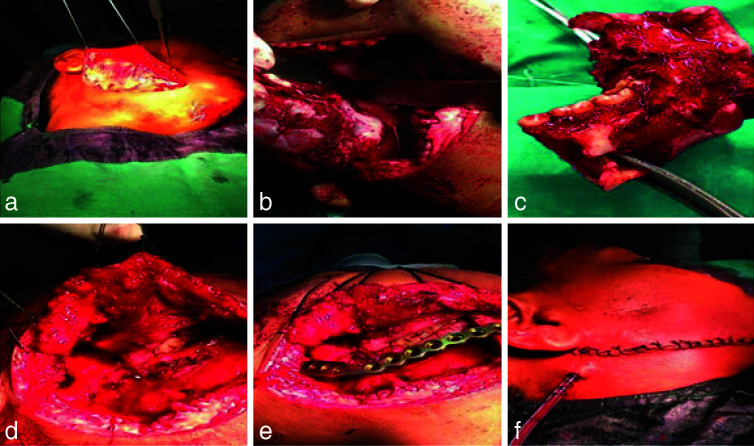
**a:** Elevation of the flap for the tumor exposure, **b:** Removal of the tumor, **c:** Gross tumor, **d:** Exposure of the site after the removal of tumor, **e:** Insertion of reconstruction plate, **f:** After suturing completion

**Figure 6:**
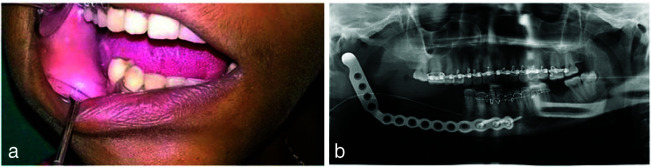
**a:** Follow up clinical picture of the patient after 6 months, **b:** Post operative radiograph of the patient

With the correlation of all clinical, histopathological, and immunohistochemical features and the presence of inflammatory cells, the final diagnosis of an intra-osseous inflammatory myofibroblastic tumor was given. The tumor was excised under general anesthesia by an extra- oral approach (Figures 5a). A hemimandibulectomy was done along with the removal of teeth numbers 44, 45, 46, 47, and 48 (Figures 5b-d). The defect was restored with a titanium reconstruction plate, and the site of the operation was closed with extra-oral suturing (Figures 5e-f). The 1-year follow-up of the patient was uneventful; no signs of recurrence were observed (Figures 6a-b). 

## Discussion

Inflammatory pseudotumor was the term used to define various histopathological entities that have spindle cell proliferation with significant chronic inflammatory cell infiltration [ 5 ].IMT was designated as one of those entities under the collective term inflammatory pseudotumor [ 6 ]. In 1994, WHO designated IMTs as a separate neoplastic entity defined as an intermediate soft tissue tumor that is composed of myofibroblasts, differentiated spindle cells, and numerous inflammatory cells, including lymphocytes, plasma cells, eosinophils, and histiocytes. Inflammatory pseudotumors are considered non-neoplastic entities that do not recur and metastasize; however, IMTs are considered tumors of intermediate grade [ 3 - 4 , 6 ].

The etiology of IMTs is still not known; however, it has been suggested that the tumor could be of autoimmune origin due to its regression with corticosteroids and cyclosporine .

With respect to its neoplastic nature, the dysregulation of the ALK gene has been noted in several cases; in the present case, the positive expression of ALK was also noted [ 8 ]. IMTs are rare in the head and neck area and exceedingly rare in the jaws. To the best of our knowledge, only 14 cases of IMTs have been reported in the jaws [ 3 , 4 , 10 ] Our case is preferably the 15th case of jaw IMT. Out of 14 published cases of Jaw IMT, 12 have been occurred in mandible and 2 have been occurred in maxilla. In gender it has been noted that jaw IMT, is more common in females with 10 reported cases than in males with 4 cases. The age distribution has been noted from the age of 7 years to 61 years with the mean age of 34.38 [ 5 - 6 , 10 - 12 ].

 Clinically, IMTs are asymptomatic and slow-growing; in the present case, the lesion was slowly enlarging in its size. Radiologically, IMTs of the jaws show insignificant features, from unilocular radiolucency to multilocular radiolucency [ 9 ]. The present case showed a multilocular lesion and was provisionally diagnosed as ameloblastoma.

Microscopically, IMTs are characterized by spindle cell proliferation in a fasicular or stroiform pattern with an admixture of various inflammatory cells infiltrates, including lymphocytes, plasma cells, eosinophils, and histiocytes. The connective tissue stroma could be myxoid, vascular, hypocellular fibrous, or hypercellular sparse [ 3 - 4 , 6 , 9 ]. The differential diagnoses include nodular fasciitis, neurofibroma fibrosarcoma, IPT, solitary fibrous tumor, and leiomyosarcoma (Table 1). Immunohistochemical profiling is of utmost importance for the final diagnosis of IMTs. 

**Table 1:** Differential diagnoses of IMT (IMT: Inflammatory pseudotumor, CD: Cluster of differentiation, ALK: Anaplastic Lymphoma Kinase) [3, 4, 10, 11]

S. NO	Differential diagnoses	Differentiating features
1	Inflammatory pseudotumors	Inflammatory pseudotumors is a group of non - neoplastic inflammatory spindle cell lesions. IMTs were previously classified under a broad term Inflammatory pseudotumor, now it is designated as a true tumor.
2	Nodular fasciitis	Nodular fasciitis generally shows C shaped fascicles with no or minimal inflammatory component.
3	Solitary fibrous tumor	Solitary fibrous tumors show large dilated vascular spaces (hemangiopericytoma like pattern) and they show positive expression for CD 34.
4	Benign Fibrous histiocytoma	Benign fibrous histiocytomas show storiform pattern and on immunohistochemistry they are negative for ALK.
5	Leiomyosarcoma	Leiomyosarcomas, show dysplastic changes with cigar shaped nuclei.
6	Myofibroma	Myofibromas, lacks inflammatory infiltrate which is one of the peculiar signs of IMT.

IMTs have been reported to show positive expression for ALK in half of the cases, with a variability of 36% to 71%. IMTs usually lead to positive results for SMA but insignificant expressions for other myeloid markers, including desmin, H-caldesmon, and transgelin. The tumor cells show no reactivity for S 100 [ 10 , 12 ].

The present case showed diffuse positivity for Vimentin and ALK, suggesting its connective tissue origin and dysregulation of the ALK gene. The tumor cells and blood vessels showed positivity for SMA, confirming its myofibroblastic origin. The tumor cells showed no reactivity to S-100, excluding the chances of neural origin. The tumor cells showed weak positivity for desmin.

The treatment of jaw IMTs varies from lesion to lesion; the present case was treated with an aggressive approach due to its multilocular, osteo-destructive radiological pattern and since the IMTs are considered inter mediate-grade tumors based on their biological behavior. None of the reported cases of oral IMT have shown a recurrence so far [ 3 - 4 , 9 , 12 ]. A signed consent form was obtained from the patient for the publication of the case report. 

## Conclusion

Jaw IMTs are rare intermediate-grade neoplasms that tend to mimic malignant tumors in their clinical and histopathological features; the proper diagnosis requires meticulous immunohistochemical profiling.
